# Thermal Changes of Root Surface of Anterior Primary Teeth in Pulpectomy with Er:YAG Laser

**Published:** 2018-05

**Authors:** Zahra Bahrololoomi, Reza Birang, Nasim Chiniforush, Hazhir Yousefshahi, Elnaz Foroughi

**Affiliations:** 1 Associate Professor, Department of Pediatric Dentistry, School of Dentistry, Shahid Sadoughi University of Medical Sciences, Yazd, Iran; 2 Professor, Department of Periodontics, School of Dentistry, Isfahan University of Medical Sciences, Isfahan, Iran; 3 Researcher, PhD of Laser Dentistry, Laser Research Center of Dentistry (LRCD), Dentistry Research Institute, Tehran University of Medical Sciences, Tehran, Iran; 4 Dentistry Student, School of Dentistry, Isfahan University of Medical Sciences, Isfahan, Iran; 5 Assistant Professor, Department of Pediatric Dentistry, School of Dentistry, Arak University of Medical Sciences, Arak, Iran

**Keywords:** Lasers, Solid-State, Pulpectomy, Tooth, Deciduous, Thermal Conductivity

## Abstract

**Objectives:**

Successful root treatment depends on elimination of microorganisms from the root canal. Considering incomplete removal of bacteria from the canal by usual methods, lasers have been suggested as a new modality. Despite their anti-bacterial properties, lasers can cause thermal changes. This study assessed the thermal changes of root surface in pulpectomy of primary teeth following the use of Er:YAG laser.

**Materials and Methods:**

Sixty primary anterior teeth were collected and prepared by K-file up to number 50. Then, they were randomly divided into two groups and were irradiated with Er:YAG laser. The first group was irradiated with 1 W laser and the second group with 1.5 W laser. The laser irradiation time was two 10-second cycles with a 2-second interval in both groups. Thermal changes were measured by a thermometer in the apical and coronal areas per second. The results were analyzed by repeated measures ANOVA considering the laser power as between-subject variable.

**Results:**

There was a temperature increase in the coronal and apical areas in use of 1 W power. There was a temperature rise in the coronal and apical areas in use of 1.5 W power. The temperature rise in the apical third was more than that in the coronal third; also, the average temperature rise was more in use of 1.5 W power than 1 W power.

**Conclusions:**

As the average temperature increase was not more than 7°C in any group, this type of laser seems to be suitable for root treatment of primary anterior teeth.

## INTRODUCTION

Early loss of primary teeth can cause malocclusion, unesthetic appearance, speech problems and temporary or permanent functional impairment. It is imperative to preserve pulp vitality as much as possible [1]. However, root canal therapy in primary teeth may be required in some cases such as irreversible pulpitis and pulp necrosis in order to prevent damage to permanent teeth [2]. In some occasions, pulpectomy alone is not sufficient [3], and cleaning, shaping and filling of the canal with absorbable pastes yields successful results [4–6].

Microorganisms are mainly responsible for root canal treatment failure [7,8]. Manual cleaning and shaping along with the use of chemical irrigating solutions may not be able to completely eliminate the microorganisms from the infected root canals [9,10]. According to Peters [11], nearly half of the canal walls remain unprepared when using the manual NiTi and stainless steel systems. Different methods such as sonic and ultrasonic equipment have been suggested to improve the root canal irrigation quality [12, 13].

Laser is another modality suggested for activation of irrigating solutions in 2009 [14]. Laser has many advantages such as no mechanical contact and not creating smear layer, and can be used for decontamination of root canal and control of bleeding [15–17]. Birang et al, [18] in their study on single-rooted permanent teeth showed that Er:YAG laser results in better smear layer removal than a simple rinse with sodium hypochlorite. Also, Noiri et al. [19] indicated the effectiveness of Er:YAG laser for elimination of endodontic pathogens. Many types of lasers are used in pediatric dentistry [20]. Soares et al. [21] showed that using Er,Cr:YSGG laser for cleaning of the root canals of primary teeth had similar canal cleaning efficacy compared to rotary instruments and was superior to manual instruments. However, some controversial results have also been reported. For instance, Liu [22] indicated higher success rate for treatment with Nd:YAG laser in comparison with formocresol. On the contrary, Odabaş et al. [23] reported that the treatment success rate with Nd:YAG laser was equal to that with formocresol. Er:YAG laser is another type of laser, which has many applications in pediatric dentistry such as enamel etching, caries removal, cavity preparation and pulp therapy [15, 24]. For instance, Kuvvetli et al. [25] evaluated contaminated primary teeth and showed stronger anti-bacterial effects of Er:YAG and diode lasers in comparison with sodium hypochlorite. Despite the aforementioned advantages for laser, laser irradiation may cause some undesirable results. The temperature increase of the tooth and pulp tissue can be one of these undesirable outcomes [26–28]. Temperature increase can cause cementum damage, root resorption, alveolar bone necrosis and pain. Zach and Cohen [29] and some other researchers reported that temperature increase by 5.5°C for one minute can cause irreversible pulpitis [30–32].

Eriksson et al. [30] concluded that temperature increase of 10°C for one minute was high enough for alveolar bone necrosis. Generally, 7°C increase in temperature is considered as the tolerance threshold for periodontal tissues [33]. Regarding the morphologic and structural differences between primary teeth and permanent teeth, they may also be different in terms of thermal changes [34, 35]. Another disadvantage of laser in dentistry could be the possibility of apical perforation of curved canals because of straight line of laser beam. According to Li et al, [36] Er:YAG laser could ablate dentin and enamel; thus, if the pulse reaches the dentin surface in a wrong angle, it could cause apical perforation; this limits the laser application to straight canals only. The effect of thermal changes caused by laser during pulpectomy of primary teeth has not yet been studied; thus, this study was conducted aiming to assess the effect of thermal changes caused by laser during pulpectomy of primary anterior teeth.

## MATERIALS AND METHODS

### Sample preparation:

This study was conducted on 115 hopeless, severely decayed, primary anterior teeth. The study protocol was approved in the ethical committee of our university (code:32085). Sixty teeth with root resorption less than one-fourth of the root length were selected. The crowns of the teeth were cut by a diamond fissure bur such that the remaining root length was 10 mm. Then, the roots were cleaned and shaped to 9 mm working length using a K-file (Kerr, Orange, CA, USA) up to number 50. During cleaning and shaping, the canals were rinsed with 5.25% sodium hypochlorite. After preparation, the teeth were autoclave-sterilized at 134°C for 15 minutes [37]. The teeth were kept in distilled water at room temperature after extraction.

*Laser irradiation and temperature measurement:* The teeth were randomly divided into two groups. They were placed in a Teflon mold with a height of 5 cm and diameter of 10 cm. A digital thermometer (ST-8891E; Standard, Hong Kong, China) with an accuracy of 0.1°C and range of 30 to 550°C, which had data logger was used. One of the thermometer probes was in contact with the root surface in the coronal third and the other probe was in contact with the apical third to measure the temperature in these areas. The other probe of the device was placed at room temperature to compare the thermal changes in root surface with the room temperature changes. The samples were irradiated with Er:YAG laser (Fidelis, Fotona, Slovenia) at a wavelength of 2940 nm, fiber length of 20 mm and diameter of 300 μ in spiral motion. Laser irradiation was started from the apical third 1 mm away from the radiographic apex and 9 mm away from the preparation endpoint. In the first group, laser with 1 W power, 100 mJ energy, 10 Hz frequency and energy density of 70.77 J/cm2 in short pulse mode (250 ms) was irradiated for 20 seconds (two 10-second cycles with a 2-second time interval). In the second group, laser with 1.5 W power, 150 mJ energy, 10 Hz frequency and energy density of 106.15 J/cm^2^ in short pulse mode (250 ms) was irradiated for 20 seconds (two 10-second cycles with a 2-second time interval). The temperature was recorded at each second, sent to the computer automatically and saved in Thermometer-E software. The results were analyzed by repeated measures ANOVA considering the laser power as between-subject variable using SPSS version 22 (SPSS Inc., IL, USA).

## RESULTS

**Fig. 1:**
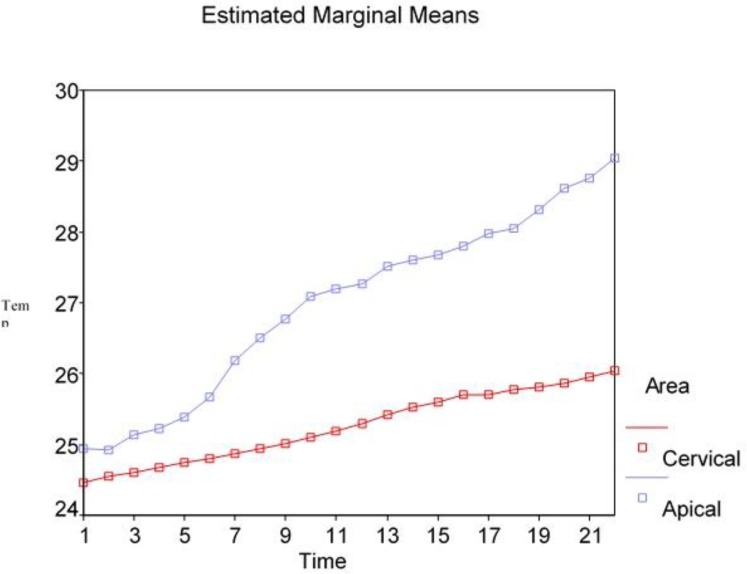
Thermal changes in the apical and cervical thirds of the root surface caused by 1 W laser when the preliminary temperature was 22°C

**Fig. 2:**
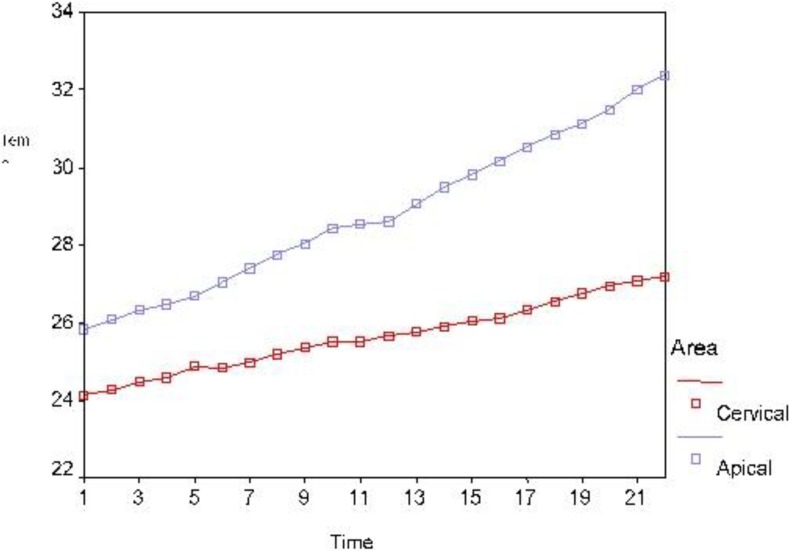
Thermal changes in the apical and cervical thirds of the root surface caused by 1.5 W laser when the preliminary temperature was 22°C

Figures 1 and 2 show the recorded temperatures from the root surface during laser irradiation (for two 10-second rounds with a 2-second time interval) with 1 and 1.5 W powers.

**Fig. 3:**
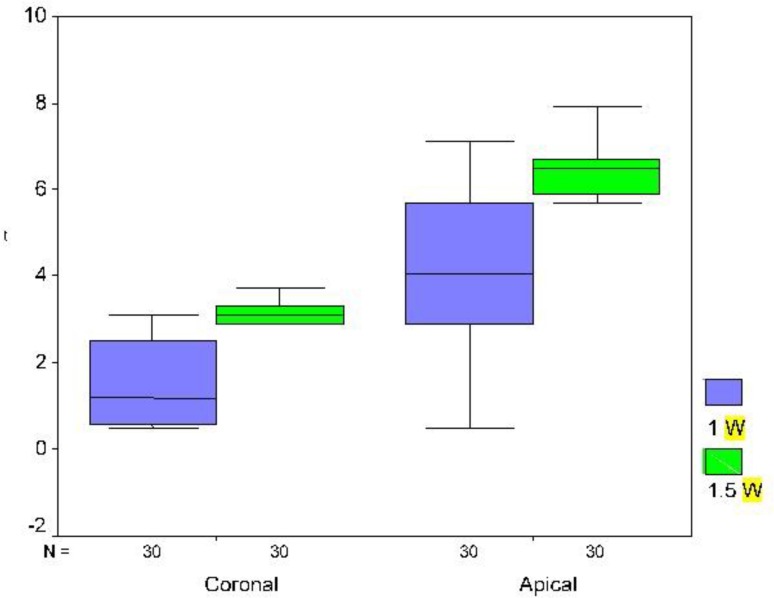
Boxplot of the mean thermal changes after 1 W and 1.5 W laser irradiation in the coronal and apical thirds

Based on the results of repeated measures ANOVA (quasi-likelihood information criteria = 84.4), irradiation zone, time and laser power had a significant effect on temperature changes (Table 1). The temperature increased by longer laser irradiation as shown in Figures 1 and 2. Table 1 shows the average temperature increase caused by 1 and 1.5 W powers of laser for 22 seconds. Based on the test results, in both tested powers, there was a significant difference between the cervical and apical thirds (P<0.001); the temperature increase in the apical third was more than that in the cervical third. Comparing 1 and 1.5 W powers based on the test results (P<0.001), the temperature increase by 1.5 W power was significantly more than that by 1 W power. The maximum temperature increase by 1 and 1.5 W laser during 22 seconds in the apical third was 7.1°C and 7.9°C, respectively (Table 1). The maximum temperature increase for 1 and 1.5 W laser during 22 seconds in the coronal third was 3.1 and 3.7°C, respectively (Table 1). Figure 3 shows the range of thermal changes during 22 seconds for the apical and coronal thirds caused by 1 and 1.5 W laser.

**Table 1.** Thermal changes in the apical and coronal thirds of the roots after applying laser.

**Output power**	**Maximum change in 22 seconds**		**Average change in 22 seconds**	

	**Coronal**	**Apical**	**Coronal**	**Apical**
1 W	3.1	7.1	1.57±0.953	4.1±1.81
1.5 W	3.7	7.9	3.04±0.45	6.52±0.63

## DISCUSSION

Success of root canal treatment is directly related to the removal of microorganisms from the root canal system [38]; but complete removal of microorganisms from the root canal system is not possible after cleaning, shaping and irrigation in all cases [39–41]. Recently, laser was introduced as a method of decontamination and removing the canal debris [9, 39–41]. Many different types of lasers are used in pediatric dentistry [20]. Besides the many advantages of laser, it may cause some undesirable effects [42].

One of these undesirable effects is the thermal damage following temperature increase [43]. It seems that these thermal effects are different based on different wavelengths of laser. For instance, Schoop et al. [44] assessed the thermal effects of Er;Cr:YSGG and Nd:YAG lasers on permanent teeth and indicated that using Er;Cr:YSGG laser in five cycles of five seconds each with a time interval of 15 seconds between each two cycles caused an average thermal increase of about 8.3±0.7°C for 1 W power and 8.7±0.7°C for 1.5 W power. Also, using Nd:YAG laser in five cycles of five seconds each with a time interval of 15 seconds between each two cycles caused an average thermal increase of about 5.4±0.6°C for 1 W power and 8.2±0.4°C for 1.5 W power. Generally, lower thermal damage by Er:YAG has been previously documented. For instance, Barcellos et al. [45] indicated that Er:YAG laser caused lower temperature increase in comparison with Nd:YAG laser. Also, Wigdor et al. [46] showed that the thermal damage caused by Er:YAG laser is less than that by Nd:YAG and CO2 lasers. Therefore, considering thermal changes, Er:YAG laser seems to be more applicable and a safer choice.

One of the effective factors to prevent thermal damage is water or air spray [9, 47, 48]. Theodoro et al. [49] concluded that using laser for 30 seconds accompanied by the use of water spray as a coolant would decrease the temperature (−2.2±1.5°C). In this study, after applying laser, temperature increased; these thermal changes may be related to the use of cooling system. But it should be noted that too much spraying of water and air causes energy absorption and reduces the ablative effect of laser [50].

As mentioned in our results section, application of Er:YAG laser caused an increase in temperature. The amount of thermal change caused by the Er:YAG laser was lower than 7°C. The periodontal tissue maximally tolerates 7°C increase in temperature [33]. Since the temperature rise was not higher than 7°C in our study, it seems that Er:YAG laser is suitable for use in pediatric dentistry even in the absence of cooling systems. In addition to the type of laser, thermal changes are different at different points of the root length. Barcellos et al. [45] showed that temperature increase in the apical third was more than that in the cervical third for 1.4 W power. Our study also showed that temperature increase in the apical third was more than that in the cervical third. This is important because in the apical region, there is only a thin layer of dentin and cementum between the root canal and periodontal ligament (PDL) and the adjacent bone [45]. However, in this study, temperature increase by the Er:YAG laser was not higher than the threshold of 7°C in the apical third.

Among the adjustable laser parameters, the average power and irradiation time have important roles in safety of clinical application of laser [45]. Based on the studies by Takeda et al, [51–53] 1–2 W power is sufficient for removing the smear layer. Also, according to Cecchini et al, [54] 1.2 W power would be enough for canal decontamination. In order to examine the thermal effect of suitable powers for removing the smear layer and canal decontamination in primary teeth, among the different laser powers, 1 and 1.5 W powers were examined in this study. According to the results of this study, temperature increase was not higher than 7°C in the samples. This shows the safety and applicability of Er:YAG laser for primary teeth in 1 and 1.5 W powers. About the duration of laser irradiation, based on the study by Soares et al, [21] increasing the duration of laser irradiation would not improve the cleaning efficacy. Determining the time required for canal decontamination in primary teeth is also important since longer duration of laser irradiation would result in higher temperature rise. More research is required on this field. Another way to prevent temperature increase in the PDL is to use laser periodically and not continuously [45]. In this study, laser irradiation was done in two 10-second cycles with a time interval of two seconds, which did not cause average temperature increase over the threshold of tissues. It should be mentioned that the temperature did not decrease in the time interval; however, different results may be obtained in the human body because of the blood vessels in the PDL [32]. Further studies are required to assess the suitable duration of laser irradiation.

## CONCLUSION

According to the results, it can be concluded that using 1 and 1.5 W powers for 20 seconds (two 10-second cycles) is safe in root canal therapy. The thermal change was 4.1±1.81°C and 1.57±0.953°C for 1 W power and 6.25±0.63°C and 3.04±0.45°C for 1.5 W power in the apical and coronal thirds, respectively.
